# Participatory needs assessment and action planning for a clinical and translational research network

**DOI:** 10.1017/cts.2020.568

**Published:** 2020-12-21

**Authors:** LaKaija J. Johnson, Jolene Rohde, Mary E. Cramer, Lani Zimmerman, Carol R. Geary, Melissa K. Tibbits, Matthew Rizzo, Paul A. Estabrooks

**Affiliations:** 1 Great Plains IDeA-Clinical and Translational Research Network, Omaha, NE, USA; 2 University of Nebraska Medical Center College of Public Health, Omaha, NE, USA; 3 University of Nebraska Medical Center College of Nursing, Omaha, NE, USA; 4 University of Nebraska Medical Center College of Medicine, Omaha, NE, USA

**Keywords:** Needs assessment, stakeholder engagement, evaluation, participatory evaluation, team science

## Abstract

The goal of this study was to assess the utility of participatory needs assessment processes for continuous improvement of developing clinical and translational research (CTR) networks. Our approach expanded on evaluation strategies for CTR networks, centers, and institutes, which often survey stakeholders to identify infrastructure or resource needs, using the case example of the Great Plains IDeA-CTR Network. Our 4-stage approach (i.e., pre-assessment, data collection, implementation of needs assessment derived actions, monitoring of action plan) included a member survey (*n* = 357) and five subsequent small group sessions (*n* = 75 participants) to better characterize needs identified in the survey and to provide actionable recommendations. This participatory, mixed-methods needs assessment and strategic action planning process yielded 11 inter-related recommendations. These recommendations were presented to the CTR steering committee as inputs to develop detailed, prioritized action plans. Preliminary evaluation shows progress towards improved program capacity and effectiveness of the network to respond to member needs. The participatory, mixed-methods needs assessment and strategic planning process allowed a wide range of stakeholders to contribute to the development of actionable recommendations for network improvement, in line with the principles of team science.

## Introduction

The National Institute of Health’s (NIH) investment in clinical and translational research (CTR) infrastructure and capacity development initiatives have been central to the advancement of scientific discovery for over a decade [1]. Clinical and Translational Science Awards (CTSAs) have emerged as a key strategy for building sustainable research capacity across the translational research spectrum, from clinical work identifying potential diagnostic, preventive, or therapeutic solutions to population health approaches applying evidence-based interventions in typical clinical or community settings [2]. Despite progress related to investments in programs like the CTSA, state-level participation in the production of NIH-funded, CTR research is significantly lower in some regions of the country when compared to others [3–7].

The National Institute of General Medical Sciences’ Institutional Development Award (IDeA) Program Infrastructure for Clinical and Translational Research (CTR) [IDeA-CTR] was established in 2011 to support the development of resources and infrastructure in states that have historically had less success competing for NIH funding [8]. The IDeA-CTR program was designed to leverage the translational capacity of biomedical and behavioral research developed through the Centers of Biomedical Research Excellence (COBRE) and IDeA Network of Biomedical Research Excellence (INBRE) programs [5]. The IDeA-CTR program led to the development of 11 regional multi-institutional networks to increase CTR in areas that have historically received lower levels of NIH funding. Similar to the CTSA program, each IDeA-CTR network includes cores – termed Key Component Activities (KCAs) – that provide resources and activities to support investigators in areas such as collaboration, professional development, community engagement, and research design [8].

IDeA-CTR networks rely on internal evaluation activities to track and monitor periodic progress towards network goals. An important component of the internal evaluation plan for emerging networks is the conduct of needs assessments to identify resources needed and assets available for the conduct of CTR from the perspective of priority stakeholders including network members, faculty/clinicians with an interest in CTR, and institutional policymakers that will benefit from CTR infrastructure enhancement. This perspective is integral to strategic resource allocation for enhanced research capacity and infrastructure. CTR infrastructure is broadly defined as the facilities, services, and resources needed to enhance the capacity to conduct research across the translational spectrum [9]. Availability of CTR infrastructure influences outcomes such as productivity and scientific breakthroughs [10,11].

Existing literature provides important insight into CTR network assessment opportunities; however, important gaps remain regarding the methods to implement participatory needs assessments that prompt CTR network outcome improvement. Typically, researchers have used either surveys or interviews to assess CTR network needs [12–16]. For example, Wiley and colleagues surveyed investigators to characterize those interested in engaging with CTR, barrier identification, and targets for professional development [12]. A limitation of published CTR network needs assessments is they fail to identify recommendations and actions to address identified needs. Additionally, there is limited information on how developing networks can use needs assessment to improve network infrastructure to facilitate translation of research into public benefit [12,17]. Further, although mixed-method and participatory approaches that engage stakeholders are recommended, few studies have incorporated these strategies [10,18,19].

Participatory needs assessment represents a promising evaluation approach for proactive identification of, and response to, the needs of CTR stakeholders [20,21]. This methodology has been shown to be effective at mitigating known challenges of participatory evaluation processes: 1) ensuring genuine participation of involved stakeholders, 2) reducing administrative burden, and 3) producing evaluative products with high-levels of utilization and quality [22,23]. The methodology also has the benefit of identifying stakeholder needs across a number of areas that are critical to the development of collaborative team science – from physical resources, to organizational process, to technolgical support – among other areas [24]. The purpose of this paper is to build on past research by describing the development and implementation of a participatory, mixed-methods needs assessment process that integrated strategic action planning for the Great Plains (GP) IDeA-CTR network.

## Case Study

**Fig. 1.**
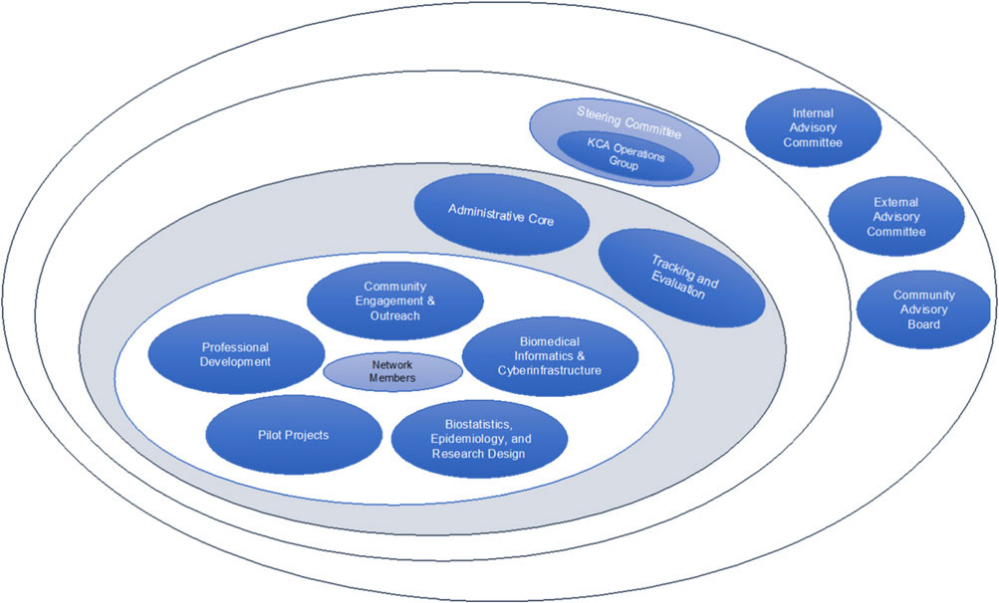
Great plains IDeA-CTR network governance ecosystem. KCA, Key Component Activities.

The GP IDeA-CTR was initiated in the fall of 2016 in direct response to the need to transform and advance clinical and translational research across Nebraska and the Dakotas by building a network for development of research systems and infrastructure (NIH NIGMS 1U54GM115458) [8]. Network members represent eight institutions, University of Nebraska Medical Center, University of Nebraska at Omaha, University of Nebraska at Lincoln, University of Nebraska at Kearney, Boys Town National Research Hospital, University of South Dakota, University of North Dakota, and North Dakota State University. Network governance structure, depicted in Fig. 1, includes internal, external, and community advisory boards, an administrative core, and a steering committee to support decision making on network activities. The steering committee is chaired by the GP IDeA-CTR principal investigator and is comprised of KCA directors (5) (Biomedical Informatics & Cyberinfrastructure: BMI-CE, Administrative Core: Admin, Tracking & Evaluation: T&E, Biostatistics, Epidemiology and Research Design: BERD, Community Engagement & Outreach: CEO, Professional Development: PD) and a representative from each partner institution.

The GP IDeA-CTR Tracking & Evaluation core was charged with implementing a formal needs assessment, with collaborative input from network stakeholders, to help inform steering committee development of strategic actions. The Institutional Review Board of the University of Nebraska Medical Center exempted the needs assessment protocol from further review. The 4-stage process included: pre-assessment, data collection, implementation of needs assessment derived actions, and ongoing monitoring of action plan progress [22] (see Table 1 and below).

**Table 1.** Stages in Great plains IDeA-CTR participatory needs assessment and action planning process

Stage	Main Activities	Participants
Stage 1: Pre-assessment	• Review literature on needs assessments in clinical and translational research networks	Administrative, T&E KCAs, Steering Committee (*n* = 26)
	• Identification of stakeholders involved in the conduct of CTR-related research at partner institutions	
	• Design of instruments	
Stage 2: Data collection, analysis, and initial actions	• Administration of survey	T&E, BERD KCAs, Steering Committee, survey respondents (*n* = 357), Characterization session participants (*n* = 75)
	• Data analysis	
	• Characterization and recommendationsessions	
	• Reporting	
Stage 3: Implementation of needs assessment derived actions	• Establishment of network priorities by steering committee	Steering committee, Admin, BMI-CE, T&E, BERD, CEO, PD, Pilot KCAs
	• Development of KCA led Action Plans	
Stage 4: Monitoring of Action Plan Progress	• Review of implementation of KCA action plans	Steering Committee, T&E KCA

BERD, Biostatistics, Epidemiology and Research Design; BMI-CE, Biomedical Informatics & Cyberinfrastructure; CEO, Community Engagement & Outreach; KCA, Key Component Activities; PD, Professional Development; T&E, Tracking & Evaluation.

### Stage 1: Pre-Assessment

The pre-assessment goal was to identify a process to provide actionable information to CTR leadership to guide network strategic program planning. Pre-assessment included defining project scope, identifying target stakeholders, and developing data collection instruments. First, the steering committee defined the scope of the needs assessment, inclusion categories for stakeholders, and the process for addressing needs within the newly formed CTR network.

A draft survey instrument was developed with questions adapted from the Rhode Island Advance-CTR Needs Assessment [12]. We customized these questions based on published CTR and/or CTSA evaluations to ensure that relevant themes were included [13,14,21]. We invited input on the draft survey from network stakeholders (e.g., network members, pilot award applicants, advisory board members).

The final 27-item survey instrument consisted of discrete and open-ended questions to assess demographic characteristics, potential barriers to respondents’ research, and types of support needed to facilitate CTR. Domain areas included data access, management, and analysis (*n* = 7); barriers and accessibility in conducting CTR (*n* = 5); educational and training offerings (*n* = 3); research resources and consultation interest (*n* = 2); satisfaction with institutional CTR efforts (*n* = 1); communication (*n* = 1); and demographics, including research area and history of funding (*n* = 8).

### Stage 2: Data collection, analysis, and initial actions

Data collection consisted of an anonymous web-administered needs assessment survey and in-person characterization and recommendation sessions. The survey developed in Stage 1 was sent to approximately 1700 faculty across the GP IDeA-CTR institutions, including all members of the CTR network (*n* = 338). To maximize response rates, surveys were disseminated by leadership at each partner institution; reminders containing the survey link were sent within one month; and a lottery incentive (five $100 gift cards) was used, as recommended by Dillman’s Tailored Design Method [25].

Approximately 27% of recipients, 457 people, initiated the survey and 357 respondents, 21%, completed the survey. Respondents were 46% female; 78% white; 16% Asian; and 4% under-represented minority. Respondents were primarily academic faculty (82%) with a relatively even split across academic rank (38% Assistant Professors; 25% Associate; 31% Professor). Thirty-two percent of respondents reported conducting biomedical/preclinical research, 32% reported completing clinical research or clinical trials while 10% and 8%, respectively were conducting population health or health services research.

Survey responses were analyzed and areas of need were identified. Respondents indicated that lack of access to data analysis support (57.1%), study participant recruitment support (55.6%), statistical expertise (55.4%), biomedical informatics equipment and/or expertise (48.8%), and access to large electronic health records/claims-based data sets (39.9%) impeded their research to at least some extent. When queried on interest in consultations, 82.6% indicated interest in having their unfunded grant applications reviewed, 80.7% were interested in data analysis, 71.3% were interested in data security and management, 69.1% were interested in study recruitment, and 66.9% were interested in biomedical informatics/data access. More than 40% of respondents also indicated that lack of access to various forms of mentoring (i.e., research, career, project, institutional, peer-to-peer) was a barrier to their research. Furthermore, results showed an uneven distribution across the translational spectrum with 32% of respondents identifying their research as biomedical/pre-clinic while only 10% engaged in population health research.

The GP IDeA CTR steering committee met to discuss survey results and define areas to be addressed in small group characterization and recommendations sessions. Three sessions addressing high-need areas as indicated by the web-based survey were planned. A fourth session addressing movement of research ideas and outcomes along the translational spectrum from pre-clinical to population health was also planned to support CTR program goals. The resultant topic areas were: 1) data analysis, 2) movement of research across the spectrum of CTR, 3) recruitment and retention of research subjects, 4) mentoring needs/support for unfunded grant applications, and 5) database design and management/data sharing and access.

The two-part characterization and recommendation sessions were hosted during the GP IDeA-CTR Annual Scientific Meeting (ASM). These sessions were designed to operate as workgroup meetings that would yield in-depth qualitative data for the characterization of each area of need development of recommendations for action. Three experts were scheduled to attend: a facilitator, an investigator with expertise in the content area, and a GP IDeA-CTR representative charged with managing applicable resources. These perspectives were considered necessary to better understand the need and to have a reliable perspective on resources available to address the need [26]. In addition, each session had a designated recorder to transcribe discussion points and recommendations. Network stakeholders interested in CTR were invited to select which session they wanted to participate in during the ASM registration. Seventy-five of the 207 ASM attendees (36%) registered for one of five sessions based on personal interest in the topic area (*n* = range of 19–41 participants). Sessions were conducted over 2 days. During the first day, participants characterized and provided more depth on the identified needs. During the second, they identified potential solutions and recommendations for action. Each group lasted approximately 1 hour per day and the same leadership and recorder were present for each to document the discussion and recommendations. A sample of the output of characterization and recommendation session is presented in Table 2.

**Table 2.** Sample need characterization and recommendations

Need	Characterization (dimensions)	Recommendations (possible solutions)
Mentoring & Support for members who have submitted grants but were repeatedly unsuccessful	• Mentoring Team Process	• External Mentoring program built around grant (e.g., 1 year grant preparation)
	• Needs continue for both mentors and mentees	• Private partnership for pilots funding – Optimize opportunities for private partnerships to fund specific requests for applications
	• Time	• Peer support group – Meet bi-monthly for collaboration, networking (current Pilot & Scholar awardees have discussed doing this); Form peer grant writing groups
	• Lack of protected time persists in multiple forms (e.g., additional work responsibilities, lack of support staff, among other)	• Mentor registry – List of People who are trained/willing to serve as mentors
	• Pilot funding/supportive infrastructure	• Preparation support for grant review (INBRE model)
	• In addition to more funding opportunities, there is a need for more support to develop competitive applications.	• Paid external review/mentoring
		• Time management – Professional Development activities built on time management that is research-specific
		• Institutional Recommendations – Allocation Model for Promotion and Tenure

INBRE, IDeA Network of Biomedical Research Excellence; RFA, Request for Application.

### Stage 3: Implementation of needs assessment derived actions

Directed content analysis – which includes starting from data collected during Stage 2 – was used to reduce the notes from each characterization and recommendation session [27]. This process allowed three investigators with qualitative data collection and analysis expertise to collaboratively generate themes related to the quantitative survey findings and recommendations that focused on resolving initial challenges to CTR that respondents identified. Using a comparison between information from the needs assessment/characterization and recommendation sessions data, 11 inter-related recommendations were identified for network action (Table 3). Recommendations were then presented to the GP IDeA-CTR steering committee to identify actionable priorities for strategic direction of the network. This prioritization activity was integrated into a regular 1-hour meeting of the steering committee and prioritization was achieved through discussion and consensus of steering committee members. During the meeting, committee members identified previous, planned, and new actions that could be applied to address recommendations. During this process, KCA(s) were assigned responsibility for action plan development based on content fit and available resources. A sample Action Plan is presented in Table 4.

**Table 3.** Directed content analysis-derived recommendations for the Great Plains (GP) IDeA CTR Steering Committee and Key Component Activities (KCAs) responsible for developing corresponding action plans

Recommendation	Responsible KCA(s)
1) Create GP IDeA-CTR Navigators Program: similar to CTSA Navigators, this team would provide support to network members throughout the collaborative research and grant preparation process.	Administrative (Admin) KCA
2) Identify and disseminate information about assets (funding, databases/registries) that exist amongst network partners that can be leveraged by members.	Community Engagement, Outreach (CEO)
	KCAs
3) Identify and address potential barriers to GP IDeA-CTR network engagement (i.e., infrastructure, resource utilization, communication, marketing).	CEO
	Admin KCAs
4) Facilitate need-specific opportunities for peer support/networking.	Professional Development (PD) Biostatistics, Epidemiology and Research Design (BERD), Biomedical Informatics & Cyberinfrastructure (BMICE) KCAs
5) Develop GP IDeA-CTR Directory/Registry: include clinicians, researchers, system partners that can be used to identify people who want to be involved in research partnerships or mentoring.	Admin,
	BMICE KCAs
6) Review GP IDeA-CTR supported professional development, and technological resource offerings and identify potential gaps.	BERD, PD
	BMICE
7) Develop a searchable repository of evidence-based interventions to help move T3/T4 research forward.	Tracking and Evaluation, CEO, PD KCAs
8) Provide support for the development of Researcher Assistant for Data (RAD) to focus on:	BERD, BMICE KCAs
a. Data availability including access to data from multiple source	
b. Tools to support researchers at all stages of research (technology to support collaboration)	
c. Database design resources	
d. Unique/custom development of databases	
9) Develop protocols for statistician and researcher collaborations that spells out agreements about authorship, time constraints, etc.	BERD KCA
10) Establish regular presentations, workshops, consultations related to participant identification, recruitment, and retention (ongoing basis).	PD, BMICE, CEO KCAs
11) Provide guidance to GP IDeA-CTR network institutions looking to adopt policies that will support translational research collaboration.	Admin KCA

**Table 4.** Sample Action Plan

Recommendation: Develop a searchable repository of evidence-based interventions to help move T3/T4 research forward							
Action Steps to Address Recommendation	Responsible Person(s)	Timeline for Completion	Intended Outcomes & Measurement	Potential Challenges/Constraints	Resources Needed	Status	Notes & Accomplishments
						1 = Enacted	
						2 = In process or under consideration	
1. Identify existing repositories	T&E Coordinator	1/31/19	List of existing repositories	Looking for actual repositories with access to existing evidence-based programs, policies, or practices. Not just summaries and reviews of existing literature)	Human	1	After reviewing existing resources, it was decided to create a webpage where researchers can find existing searchable repositories (e.g., NCI’s RTIPs).
2. Develop categories for existing interventions	T&E Coordinator	2/14/19	Outline for the web-based repository	None	Human	1	Many resources have already been categorized (e.g., behavioral health, substance abuse, etc.) and can be displayed as such on the website.
3. Create an evidence-based intervention resource page that links to existing repositories	T&E Coordinator/BMICE Coordinator	3/14/19	Webpage link on network website	Getting the programming completed might be a challenge	Human resources from BMICE and TE	1	We worked with the BMI Coordinator to display the evidence-based interventions resource page on GP IDeA CTR Website. The evidence-based repository page was shared as part of a breakout session at Annual Scientific Meeting 2019, “Evidence-Based Intervention and Comparative Effectiveness Research,” led by T&E Director. Attendees were encouraged to share recommendations for resources to be added. An attendee requested the list of evidence-based repositories be shared with local health department directors.
4. Survey membership for evidence-based interventions that have been locally developed or are being implemented	T&E Coordinator/ CEO Coordinator	Summer 2019	Repository of local evidence-based interventions	Survey response	Human	2	The IDeA-CTR membership was surveyed (March/April 2019) on their interest in joining a Dissemination and Implementation (D&I) Science interest group. D&I interest group will be surveyed to identify local evidence-based programs that will be added to our website resource page.
5. Develop category of locally developed and implemented evidence-based interventions for the website	T&E Coordinator/BMICE Coordinator	Summer 2019	Webpage link on Center website	Getting the programming completed might be a challenge	Human resources from BMICE and T&E	2	
6. Identify scientists within the network that can consult on selection of evidence-based interventions and implementation evaluation	T&E Coordinator/T&E Director	Summer 2019	List of consultants for D&I research (T3/T4)	Identifying folks	Human	2	We will identify a list of consultants for this work from the D&I interest group (*n* = 83).

BMICE, Biomedical Informatics & Cyberinfrastructure; CEO, Community Engagement & Outreach; D&I, Dissemination and Implementation; GP, Great Plains; NCI, National Cancer Institute; RTIP, Research Tested Intervention Program; T&E, Tracking & Evaluation.

### Stage 4: Monitoring action plan progress

The Tracking & Evaluation core reviewed each action plan and followed up with each KCA on areas in need of clarification or further specification. A process for ongoing action plan monitoring was integrated into the existing quarterly reporting process for KCA communication to network leadership regarding activities toward KCA aims. Quarterly meetings allow core directors to discuss progress towards completion of aims and to report on revisions to proposed KCA activities and assess areas where additional resources may be required. KCA directors recorded successes and challenges in implementing action items in the “Notes & Accomplishments” column of the Action Plan template as seen in Table 4. The sample action plan details steps taken to develop a web-based list of Tools for Identifying, Selecting, and Implementing Evidence-Based Interventions (available online at: https://gpctr.unmc.edu/ctr-resources/tools-for-identifying-selecting-and-implementing-evidence-based-interventions/). It also highlights the opportunity to report on collaboration across KCAs to implement recommendations and report benefits derived by network members.

## Discussion

Participatory needs assessment processes represent an important opportunity for continuous improvement of developing CTR research networks [20,21].The goal of this process was to provide actionable information to the GP IDeA-CTR Administrative KCA, KCA Directors, and institutional partners for strategic action planning to strengthen resources and services for clinical and translational research across the network. The participatory, mixed methods process provided clarification and additional insights into the strengths and areas for improvement identified in the needs assessment survey. From this work, we are able to make four generalizations. First, the needs of an emerging CTR network appear to be similar to those experienced by other networks, both new and established. Second, the mixed methods approach to engagement of multiple levels of stakeholders resulted in clarification of needs beyond what was possible using a single survey approach. Third, the blending of member and steering committee interests encouraged prioritizing actions in support of network aims, but previously unplanned. Fourth, integration of the needs assessment process within the ongoing GP IDeA CTR network governance structure provided opportunity for action specification to resolve needs and accountability to address needs in a timely manner.

Understanding and addressing barriers to conducting CTR research in states with historically low federal funding is a key to more fully leveraging federal investments aimed at enhancing research capacity and infrastructure. Despite being in a state with IDeA designation, needs of the participants engaged in this assessment were not different than obstacles described in the literature [12,13,15]. Some of the needs identified in this process, that is, support for team science in promotion and tenure, are not directly influenceable by the CTR network and require institutional attention[21]. This highlights opportunities for CTR networks to advocate for changes to institutional processes and policies that may enhance participation in translational science.

When compared to previous CTR network-related needs assessments, our approach used a more inclusive participatory process to generate a deeper understanding of identified needs and developed action plans to resolve barriers. For example, lack of member clarity regarding resources available to GP CTR members and housed locally at partner institutions was an important finding. The needs assessment and action planning process allowed the KCA leadership to identify, promote, and leverage access to various human, equipment, and funding resources that exist within and across the GP CTR partner institutions, network members, community, and industry partners. The multi-step process described will continue to allow the GP CTR network to collaboratively implement action plans that target investments towards innovative programs and services that will increase communication, awareness, and access to network CTR infrastructure [11].

Steering committee prioritization of recommendations based on potential for action was a novel aspect of our needs assessment process. KCAs establish priorities annually based on tasks required to achieve network aims. These priorities are reviewed quarterly and can shift during the year based on the provision of service to network members and constraints (time/resources). The prioritization activity allowed KCA leadership to develop detailed action plans that identified misalignment of proposed activities with yearly goals and objectives as well as resources needed for implementation. This also provided KCA leadership with an opportunity to communicate action plan progress transparently. The development of a process to respond to the needs of CTR stakeholders and KCA leadership is integral to our ability to enhance our network’s success in facilitating the movement of research ideas and findings more efficiently across the translational research spectrum.

There are some limitations to our approach. One is that our process for the characterization and recommendation sessions allowed members to identify and contribute to the needs that they perceived to be most relevant. However, by self-selecting to personally relevant sessions, there is potentially an opportunity lost. Session attendees with different perspectives could have led to different characterization and recommended actions. We viewed the benefit to outweigh the costs of this approach. Another limitation is that we didn’t have comparative data to determine the representativeness of our sample to the broader population of clinical and translational research stakeholders in our network. Future work would be improved by documenting representativeness and potentially examining needs by investigator rank.

The Tracking and Evaluation KCA’s first aim was to evaluate the GP CTR network’s leadership and governance activities. Effective leadership and governance are essential to the success of scientific consortia [28]. Network leadership plays a key role in the ability of the network to achieve CTR goals not attainable through individual institutional efforts [29]. Given the dynamic nature of CTR networks, it is important that their organizational structures reflect the ongoing needs and evolving perspectives of members and stakeholders. Incorporating reporting progress towards completion of action plans has proven to be an effective method to identify governance structures and processes that may facilitate or inhibit proposed actions. The described process is in alignment with achieving NIH strategic goal of promoting a culture of accountability and good stewardship across CTR networks [30] and represents an opportunity to bridge gaps in evaluation and continuous improvement through accountability.

## Conclusion

Assessing and prioritizing action to reduce barriers to the conduct of CTR is essential to the development of research capacity and infrastructure in IDeA states. The needs assessment process developed for the GP IDeA-CTR provided the opportunity for a wide range of stakeholders to contribute to the identification of research impediments and development of recommendations. This, in turn, allowed CTR leadership to translate findings to strategic planning and action to improve achievement of network aims using previously unidentified strategies. This process also demonstrated progress towards the adoption of a network-wide culture of shared governance and team science, reflected by the adoption of a less hierarchical organizational structure and broad engagement of stakeholders to determine action needed to improve access to key resources for the conduct of CTR. Participatory approaches for continuous improvement will be essential to our network’s long-term sustainability and the development of programs and policies to achieve our ultimate goal of enhancing capacity for multi-institutional team science and CTR in the region.
